# Clinical characterization of necrotizing enterocolitis in neonates with or without congenital heart disease: a case–control study

**DOI:** 10.1186/s13052-025-01928-6

**Published:** 2025-03-24

**Authors:** Kaiyishaer Balati, Zhuoming Xu, Limin Zhu, Xiaolei Gong

**Affiliations:** 1 Department of Neonatology, Second People’s Hospital of Kashi Prefecture, Kashi, 844099 Xinjiang Province China; 2 Cardiac intensive care unit, Department of Cardiothoracic Surgery, Shanghai Children’s Medical Center affiliated to Shanghai Jiao Tong University School of Medicine, Shanghai, 200127 China

**Keywords:** Neonates, Preterm infant, Congenital heart disease, Necrotizing enterocolitis

## Abstract

**Background:**

This study aimed to analyze the clinical characteristics and differences between neonates with congenital heart disease (CHD)–related neonatal necrotizing enterocolitis (NEC) and those with non-CHD–related neonatal NEC.

**Method:**

This retrospective study included patients with CHD who met Bell’s staging diagnostic criteria and were hospitalized between 2014 and 2023 in the Cardiac Intensive Care Unit of Cardiothoracic Surgery, Shanghai Children’s Medical Center affiliated to Shanghai Jiao Tong University School of Medicine. These patients comprised the CHD-related NEC group (CHD-NEC group). Meanwhile, the control group included randomly matched non-CHD–related NEC children (nCHD-NEC group) admitted to the neonatal department of the Second People’s Hospital in Kashi Prefecture according to the 1:2 matching principle. Patients’ basic information, adverse clinical events before NEC onset as well as NEC severity and treatment were recorded.

**Results:**

The CHD-NEC group comprised 60 cases, whereas the nCHD-NEC group comprised 120 cases. Compared with the nCHD-NEC group, the CHD-NEC group had an older gestational age (38.71 [37.89, 39.67] weeks vs. 30.65 [29.68, 32.29] weeks, *p* < 0.001); a heavier birth weight (3.2 [2.69, 3.67] kg vs. 1.39 [1.1, 1.59] kg, *p* < 0.001); and higher proportions of patent ductus arteriosus (73.3% vs. 26.7%, *p* < 0.001), shock (81.7% vs. 36.7%, *p* < 0.001), and mechanical ventilation requirement (91.7% vs. 51.7%, *p* < 0.001). At disease onset, the CHD-NEC group had a higher vasoactive drug score (16.75 [7.26, 23.63] vs. 0 [0, 10], *p* < 0.001) but lower values for the proportion of infants who were small for gestational age (15% vs. 33.3%, *p* = 0.045), incidence of premature rupture of membranes (3.3% vs. 26.7%, *p* = 0.002), incidence of early onset sepsis (6.7% vs. 23.3%, *p* = 0.038), and incidence of late onset sepsis (46.7% vs. 70%, *p* = 0.036) than the nCHD-NEC group. Among children who required abdominal surgery, the CHD-NEC group tended to have more colon involvement (6.6% vs. 0.8%, *p* = 0.063), but no significant difference in mortality was noted between the two groups.

**Conclusion:**

Children with CHD-NEC and nCHD-NEC have significantly different clinical characteristics. CHD-NEC is mainly observed in full-term infants with appropriate weight for gestational age, and perioperative intestinal ischemia may be the main pathophysiology. Conversely, nCHD-NEC is mainly noted in preterm infants, possibly related to immature intestinal development and infection. Large prospective clinical research is warranted to explore the pathogenesis, pathophysiology, indicator monitoring, and treatment plan for children with NEC.

## Introduction

Neonatal necrotizing enterocolitis (NEC) is a serious gastrointestinal complication among newborns in the neonatal intensive care unit (NICU), usually causing intestinal necrosis and multiple organ failure [1]. Its classic form is mostly observed in preterm infants treated in the NICU, especially those with a very low birth weight (< 1500 g) [2]. However, it could also occur in term infants suffering from congenital heart disease (CHD) with hemodynamic instability [3, 4]. The pathogenesis of NEC in premature infants may be different from that in term infants. Perhaps, the exacerbation of preterm status contributes to the intestinal immune insufficiency and flora disorder [1, 5], and the pathophysiology of NEC in children with CHD might be mainly related to systemic hypoperfusion, which causes ischemia and hypoxia of mesenteric vessels [6, 7]. Currently, the mortality rate of NEC requiring surgical intervention is as high as 20–30% [6–8].

Although the clinical manifestations of CHD-related NEC (CHD-NEC) and non-CHD–related NEC (nCHD-NEC) are relatively similar, further exploring their differences in pathogenesis, onset time, severity, surgical intervention requirement, and mortality is still necessary. Hence, this study aimed to review and analyze the clinical differences between the CHD-NEC and nCHD-NEC groups, summarize their occurrence and development, and facilitate more refined preventive management and treatment in children in the future.

## Methods

### Participants

**Fig. 1:**
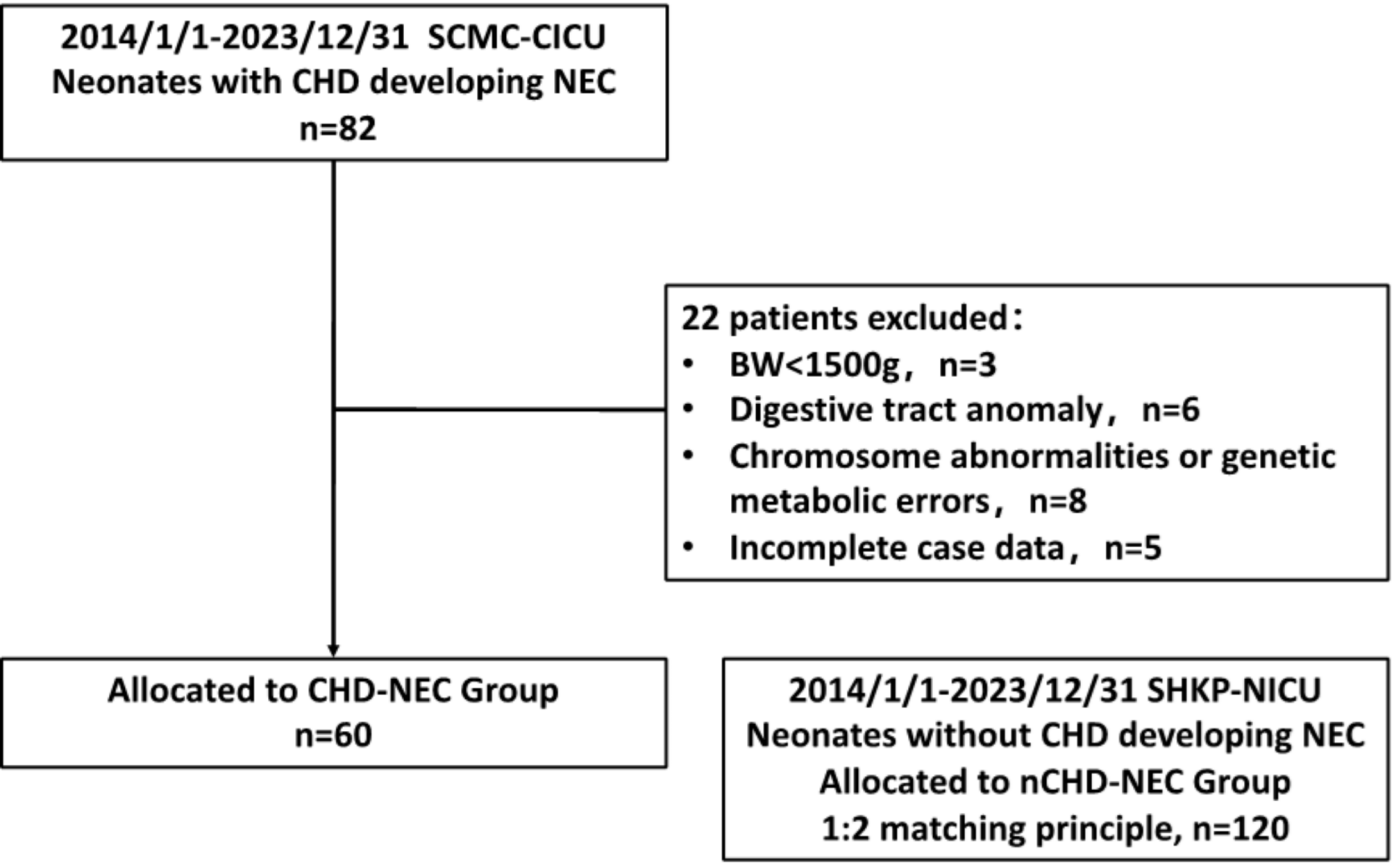
Flowchart of the screening process. Note: BW, body weight; SCMC-CICU, Shanghai Children’s Medical Center–Cardiothoracic Surgery Intensive Care Unit; SHKP-NICU, Second People’s Hospital of Kashi Prefecture–Neonatal Intensive Care Unit

This 1:2 retrospective case–control study recruited neonates with critical CHD who developed NEC, met Bell’s staging criteria [9], and were hospitalized between January 1, 2014 and December 31, 2023, in the Cardiothoracic Intensive Care Unit (CICU) of Shanghai Children’s Medical Center affiliated to Shanghai Jiaotong University School of Medicine. The exclusion criteria were as follows: (1) body weight < 1500 g with CHD (could not be classified in this study); (2) congenital anomaly of the digestive tract as a comorbidity; (3) incomplete case data; and (4) chromosome abnormalities or genetic metabolic errors. Sixty patients with NEC met the inclusion criteria, constituting the CHD-NEC group. As for the control group, patients were selected from the Department of Neonatology of the Second People’s Hospital of Kashi Prefecture, an alliance unit of Shanghai Children’s Medical Center; according to the 1:2 matching principle of the number of cases, 120 children with nCHD-NEC were randomly matched using SPSS software at the same time. Since there are relatively few CHD-NEC pediatric patients, using a 1:2 ratio can include as many CHD-NEC and nCHD-NEC patients as possible while ensuring a sufficient sample size to improve the representativeness of the study. Figure 1 shows the patient flowchart of screening for inclusion.

### Ethical considerations

This retrospective study was conducted in accordance with the Declaration of Helsinki and was obtained approval from the Ethics Committee of Shanghai Children’s Medical Center affiliated to Shanghai Jiaotong University School of Medicine (No. SCMCIRB-K2023026-1) and the Second People’s Hospital of Kashi Prefecture (No. [2024]-LKS-24). The need for informed consent was waived by the Ethical Committee of these two centers due to the study’s retrospective nature. This study is supported by “SJTU Trans-med Awards Research” (No: 20220101 to Zhuoming Xu).

### Data collection

This study collected children’s general information such as gestational age, birth weight, gender, 1-minute Apgar score, 5-minute Apgar score, birth mode, multiple births, and small for gestational age (SGA). For patients in CHD-group, the diagnosis of CHD was collected. The following data on adverse clinical events before NEC onset were also collected: premature rupture of membranes, patent ductus arteriosus (PDA), age at NEC onset, shock with various types, vasoactive inotropic score (VIS) [10], feeding mode, red blood cell infusion and infusion times, mechanical ventilation, sepsis, and umbilical vein catheterization. Moreover, data on Bell’s stage, intestinal perforation, abdominal surgery, and mortality were obtained to assess NEC severity. Meanwhile, time of enteral nutrition (EN) after NEC treatment, time to restore oral nutrition after NEC treatment and duration of parenteral nutrition (PN) were also collected. The two center utilized the same formula of PN based on the guideline of pediatric parenteral nutrition [11].

### Statistical methods

SPSS 26.0 statistical software package (IBM Corporation, Armonk, NY, USA) was used for statistical processing. All statistical data were analyzed using two-sided tests, and type I errors were controlled to within 0.05. Normally distributed data are presented as the mean ± standard deviation to describe the difference between groups. Groups were compared using an independent samples *t*-test. For non-normally distributed data, the median (Q25, Q75) was used, and groups were compared using the Mann–Whitney *U* test. In addition, the count data are expressed as frequency (percent), and Pearson’s chi-square test was employed for group comparisons. When the theoretical frequency of > 25% of the cells was < 5 and > 1, the continuous correction chi-square test was used; for < 1, the Fisher’s exact probability method was used.

## Results

### General characteristics

The gestational age and birth weight were significantly lower in the nCHD-NEC group than in the CHD-NEC group. Most of the patients in the CHD-NEC group were boys (63.3% vs. 36.7%, *p* = 0.004). The 1- and 5-minute Apgar scores significantly differed between such groups, and the proportion of infants who were small for gestational age was significantly higher in the nCHD-NEC group than in the CHD-NEC group (33.3% vs. 15%, *p* = 0.045). In the nCHD-NEC group, 19 (15.8%), 68 (56.7%), and 22 (18.3%) of the infants had extremely low, very low, and low birth weights, respectively. Table 1 lists the general characteristics of the two groups.

**Table 1:** Comparison of the general characteristics between the two groups

	nCHD-NEC group (*n* = 120)	CHD-NEC group (*n* = 60)	Statistical values	*P* value
Gestational age (weeks)	30.65(29.68,32.29)	38.71(37.89,39.67)	37.37	< 0.001
BW (kg)	1.39(1.1,1.59)	3.20(2.69,3.67)	45.00	< 0.001
Gender, male (n, %)	44(36.7%)	41(68.3%)	8.22	0.004
Birth mode, cesarean section (n, %)	101(84.2%)	47(78.3%)	0.75	0.245
Multiple births (n, %)	22(18.3%)	7(11.6%)	3.49	0.083
1 min Apgar	7(6,9)	8(5,8)	17.56	< 0.001
5 min Apgar	9(8,10)	8(8,9)	7.44	0.01
SGA infants (n, %)	40(33.3%)	9(15%)	4.04	0.045

Note. Continuous data are presented as median (interquartile range). Categorical data are presented as counts (%). BW, body weight. SGA, Small for gestational age

**Fig. 2:**
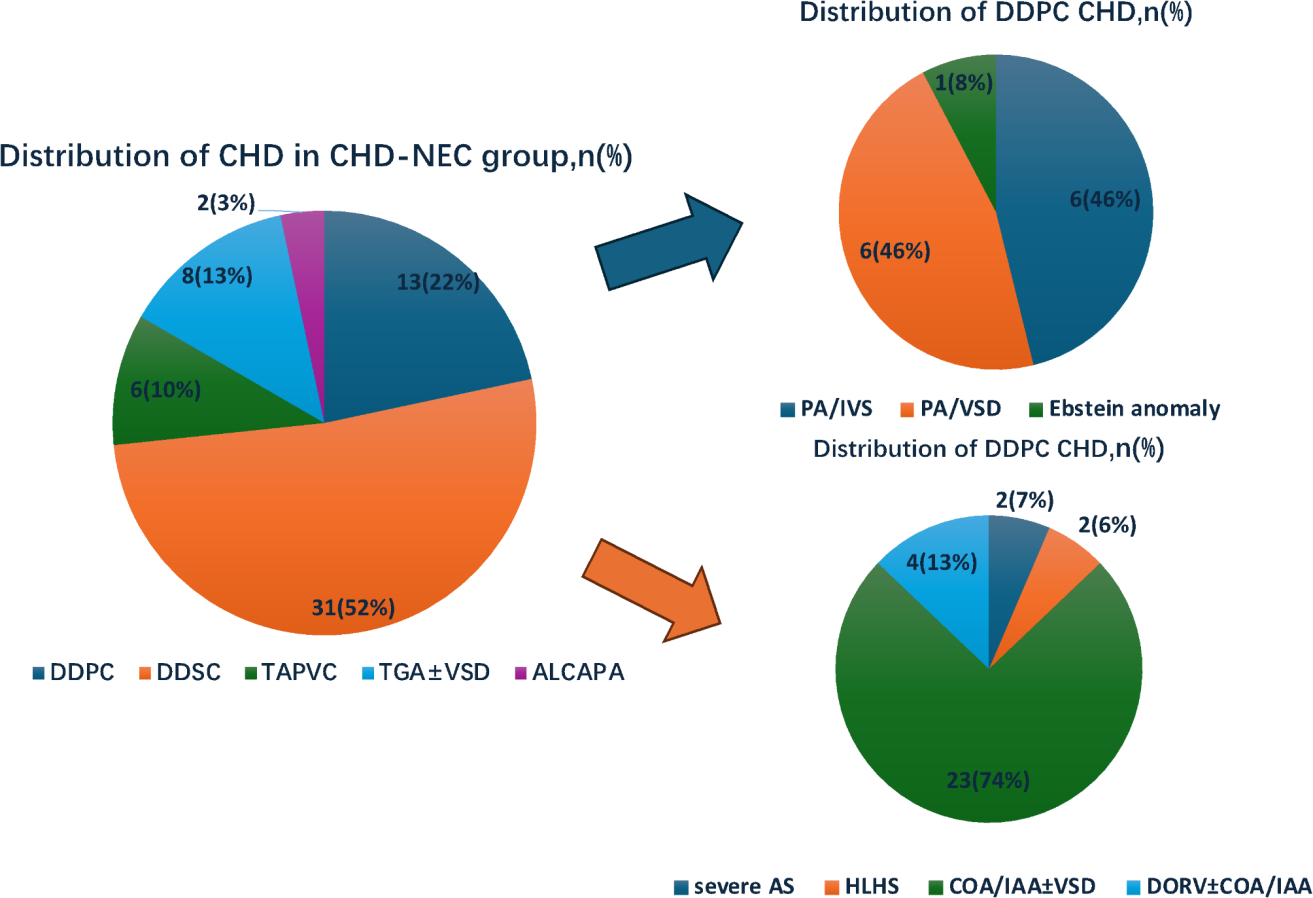
CHD classification of children with CHD-NEC. Note: DDPC, ductus-dependent pulmonary circulation; DDSC, ductus-dependent systemic circulation; TGA, transposition of the great arteries; VSD, ventricular septal defect; TAPVC, total anomalous of pulmonary venous connection; ALCAPA, anomalous of left coronary artery arising from pulmonary artery; PA/IVS, pulmonary atresia with intact ventricular septum; PA/VSD, pulmonary atresia with ventricular septal defect; AS, aortic valve stenosis; HLHS, hypoplastic left heart syndrome; COA, coarctation of the aorta; IAA, interruption of aortic arch; DORV, double outlet of right ventricle

Furthermore, Fig. 2 illustrates the CHD classification of children with CHD-NEC.

### Adverse clinical events before NEC onset

The incidence of PDA, circulatory shock, and mechanical ventilation use before NEC was significantly higher in the CHD-NEC group than in the nCHD-NEC group. The VIS was also significantly higher in the CHD-NEC group. After shock type classification, the incidence of cardiogenic shock, obstructive shock, and mixed shock was significantly higher in the CHD-NEC group than in the nCHD-NEC group. However, the nCHD-NEC group had a high incidence of premature rupture of membranes, sepsis, and several red blood cell (RBC) transfusions before the NEC onset. Regarding feeding, 28 children (23.3%) in the nCHD-NEC group were breastfed (pure breastfeeding plus mixed feeding); this number was significantly higher than that in the CHD-NEC group. In NEC severity analysis by Bell’s stage, none of the breastfed patients but 16 formula-fed patients developed IIIa or IIIb NEC (*p* = 0.443). Moreover, age at NEC onset did not significantly differ between the two groups, but when the CHD-NEC group was divided into the preoperative onset group (*n* = 15) and postoperative onset group (*n* = 45), the NEC onset age was significantly lower in the preoperative onset group than in the postoperative onset group (7 days [6, 12] vs. 16 days [12, 21], *p* = 0.025]. Table 2 details these results.

**Table 2:** Comparison of adverse clinical events before NEC onset between the two groups

	nCHD-NEC group (*n* = 120)	CHD-NEC group (*n* = 60)	Statistical values	*P* value
Premature rupture of membranes (n, %)	32(26.7%)	2(3.3%)	11.03	0.002
PDA (n, %)	32(26.7%)	44(73.3%)	19.30	< 0.001
Age at onset (days)	14.5(12.75,20.75)	14(9,21)	0.09	0.765
Circulatory shock (n, %)	44(36.7%)	49(81.7%)	18.23	< 0.001
Distributive shock	44(36.7%)	16(26.7%)	0.95	0.329
cardiogenic shock	8(6.7%)	38(63.3%)	26.01	< 0.001
obstructive shock	0	15(25%)	9.00	0.003
Mixed shock	8(6.7%)	16(26.7%)	5.00	0.025
Vasoactive inotropic score	0(0,10)	16.75(7.26,23.63)	17.46	< 0.001
Feeding (n, %)				
breast milk	4(3.3%)	0(%)	2.02	0.333
Formula	92(76.7%)	60(100%)	15.18	< 0.001
Mixed feeding	24(20%)	0(0%)	12.86	< 0.001
RBC transfusion (n, %)	96(80.0%)	49(81.7%)	0.04	1.000
Number of erythrocyte transfusions	2(2,4)	1(1,1.25)	26.46	< 0.001
Infusion of red blood cells 48 h before onset (n, %)	73(60.8%)	37(61.7%)	0.24	0.943
Mechanical ventilation (n, %)	62(51.7%)	55(91.7%)	20.09	< 0.001
Early onset sepsis (n, %)	28(23.3%)	4(6.7%)	5.18	0.038
Late onset sepsis (n,% )	84(70.0%)	28(46.7%)	4.39	0.036
Umbilical vein catheterization (n, %)	8(6.7%)	0(0%)	4.09	0.109

Note. Continuous data are presented as median (interquartile range). Categorical data are presented as counts (%). PDA, patent ductus arteriosus; RBC, red blood cell

### NEC severity and outcome

In both the CHD-NEC and nCHD-NEC groups, the proportions of Bell’s stages I and II were relatively high, showing no significant difference between such groups. Intestinal perforation and mortality also did not significantly differ between such groups. Children requiring abdominal surgery in the CHD-NEC group were more likely to develop colon involvement than those in the nCHD-NEC group (6.6% vs. 0.8%, *p* = 0.063). Meanwhile, patients in CHD-NEC group got later restarting of enteral nutrition or oral feeding than patients in nCHD-NEC, also needed longer support of parenteral nutrition, as shown in Table 3.

**Table 3:** Comparison of NEC severity and outcome between the two groups

	nCHD-NEC group (*n* = 120)	CHD-NEC group (*n* = 60)	Statistical values	*P* value
Bell Stages (n,%)			3.06	0.691
Ia	12(10%)	13(21.7%)	1.86	0.172
Ib	36(30%)	17(28.3%)	0.03	0.869
Ia + Ib	48(40%)	30(50%)	0.80	0.37
IIa	40(33.3%)	13(21.7%)	1.43	0.232
IIb	12(10%)	6(10%)	0.00	1
IIa + IIb	52(43.3%)	19(31.7%)	1.19	0.276
IIIa	16(13.3%)	7(11.7%)	0.05	0.82
IIIb	4(3.3%)	4(6.7%)	0.42	0.661
IIIa + IIIb	20(16.7%)	11(18.3%)	0.04	0.845
Intestinal perforation (n,%)	4(3.3%)	4(6.7%)	0.42	0.661
Abdominal surgery (n,%)	8(6.7%)	6(10%)	0.02	0.896
Involving only the small intestine	5(4.2%)	1(1.7%)	2.94	0.138
Involving only the colon	1(0.8%)	4(6.6%)	4.38	0.063
The small intestine and colon are both involved	2(1.7%)	1(1.7%)	0.14	1.000
Time of EN after NEC treatment (days)	11.5(8, 18.3)	14.5(9,19)	2.05	0.04
Time to restore oral nutrition after NEC treatment (days)	14(9.8, 21.3)	19(11,27)	2.83	0.005
Duration of PN (days)	18(11,24)	23(14.8,33)	3.29	0.001
Death (n,%)	12(10%)	10(16.7%)	0.72	0.532

Note. Categorical data are presented as counts (%)

## Discussion

Although the clinical manifestations of CHD-NEC and nCHD-NEC are similar, the pathophysiological mechanisms of occurrence and development may be different. The pathogenesis of CHD-NEC is mainly caused by mesenteric ischemia resulting from cardiac malformation and low cardiac output (LCO) and/or shock [12, 13]. In a healthy, fasting state, the digestive tract may require only < 5% of the total cardiac output (CO), but after feeding, the demand would increase to 30% to meet the metabolic activities of absorption and digestion of nutrition [6]. However, the bowel perfusion of neonates with CHD may not respond appropriately to this increased perfusion demand. Ductus-dependent CHD, whether ductus-dependent pulmonary circulation or ductus-dependent systemic circulation, caused by the instability of arterial ductus blood flow, usually leads to mesenteric hypoperfusion and develops NEC [14, 15]. In addition to ductus-dependent CHD, some obstructive diseases, such as total anomalous pulmonary venous connections (TAPVC), may represent reverse blood flow of the descending aorta in the diastole phase, causing diastolic mesenteric ischemia [16]. In the current retrospective study, most CHD-NEC cases were ductus-dependent CHD and TAPVC, which is basically consistent with the high-risk factors of NEC reported in the literature. The incidence of shock in children with CHD-NEC was relatively high, with the majority being cardiogenic shock (63.3%), further suggesting that CHD-NEC is mainly related to systemic hypoperfusion [17, 18]. Furthermore, the use of vasoactive inotropic drugs, especially high-dose vasoconstrictors, is related to NEC occurrence [7, 12]. The sensitivity of intestinal circulation to endogenous and exogenous catecholamines increases in the LCO state postoperatively, leading to local ischemia, which, in turn, results in the development of intestinal inflammation and endotoxemia; consequently, multiple organ dysfunction and even death may occur [8, 14]. In the current study, owing to myocardial inhibition after cardiopulmonary bypass, many neonates with CHD needed vasoactive inotropic drugs to maintain the perfusion of crucial organs, but this drug type also increased the risk of developing NEC for children with CHD-NEC. Thus, cardiac surgery is also a high-risk factor for NEC. Moreover, patients with congenital heart diseases may also suffered of other combined disorder, such as dysmorphogenetic injuries affecting the vascular supply to bowel, especially in subjects showing midline defects. These may be further crucial risk factor of NEC development. Serra et al. reported, indeed, many genetic diseases which may be related to NEC or other gastrointestinal disorders [19–22]. In our study, patients with gene abnormalities were excluded to reduce the statistical bias, but such infants should be considered as high-risk of NEC onset.

However, most children in the nCHD-NEC group were preterm, low-weight, and small-for-gestational-age infants, which all accounted for a high proportion; thus, nCHD-NEC may be related to the immature intestinal development of preterm infants. The intestinal flora in preterm infants was reported to be significantly less diverse than that in term infants [23, 24]; hence, the intestinal mucosal barrier of preterm infants may be more vulnerable to damage and NEC development. Infection is another risk factor for nCHD-NEC. The skin and mucosa of premature infants are too fragile, thereby highly at risk for injury during hospitalization; once the skin is injured, pathogens may invade, possibly leading to systemic infection [25]. Especially in septicemia in children, by the influence of multiple factors, including inflammatory factors, bacterial toxins, and anti-infective medications, the incidence of NEC can significantly increase [23, 26]. In the present study, the incidence of early- or late-onset sepsis was significantly higher in the nCHD-NEC group than in the CHD-NEC group, suggesting that neonatal sepsis is a crucial factor in nCHD-NEC occurrence and development. The incidence of NEC in neonates with sepsis is thrice that in neonates without sepsis [25].

Breastfeeding has been proven to be a protective factor against NEC in preterm infants. Its mechanism may be related to the fact that human milk contains high levels of variant globulins, growth factors, oligosaccharides, and probiotics. Therefore, preterm infants should try to breastfeed [27]. The current study shows that the severity of NEC in breastfed children is relatively low and that no children develop stage III NEC; however, the difference was not statistically significant, possibly related to the small sample size. The protective effect of breast milk on the intestinal tract of newborns with CHD warrants further study, with some reports supporting this view [28]. However, the feeding of infants with CHD is usually complex, and the slow growth, long hospitalization, and high calorie demand limit the application of breast milk in this population [17, 18, 28]. Although exclusive breastfeeding without fortification is associated with a significantly reduced risk for NEC before complex CHD surgery [28], the overall risk of developing NEC in children with CHD is high [29, 30]; thus, preoperative breastfeeding remains controversial for infants with cardiac NEC [28, 31]. Nonetheless, with the establishment of breast milk banks all over the country, the question of whether children with CHD can benefit from breastfeeding will eventually be solved. RBC transfusion may also cause NEC [32]. In particular, NEC occurring within 48 h of blood transfusion is defined as transfusion-related NEC [33]. In the present study, the need for RBC transfusion before onset and RBC transfusion 48 h before onset showed no statistically significant difference between the two groups, but the nCHD-NEC group required multiple RBC transfusions compared with the CHD-NEC group, contributing to more RBC demand in patients with infection. In addition, anemia is a risk factor for NEC. However, blood transfusion, anemia, and blood loss are mutually causal and complex; further detailed research is needed to clarify the principle [34]. Thoroughly understanding the blood transfusion indications and minimizing the extra use of blood products and blood loss are crucial for both groups and for all newborns. Studies in the literature about the role of probiotics such as Bifidobacterium bifidum, in reducing the risk of NEC among, preterm infants are contrasting [35,^36^]. In our study, because Bifidobacterium bifidum was not available in the two centers, we could not give the suggestion of probiotics utilization.

NEC in term infants is mainly located in the proximal colon, whereas that in preterm infants is mainly located in the distal ileum and ileocecum [37]. Pediatric CHD-NEC requiring abdominal surgery intervention is more likely to involve the colon [38, 39]. Bubberman et al. reported that the reason why some children with CHD-NEC are more vulnerable to ischemic damage in the colon in the “watershed area” is the phenomenon of blood theft in the diastolic period of systemic circulation [38]. However, Cozzi et al. found that the small intestine of children with CHD-NEC and nCHD-NEC was easily affected (33% vs. 31%) [40]. In the present study, children with CHD-NEC seemed to exhibit an increasing trend of colon involvement, but no significant difference was noted, possibly because of the low number of children undergoing abdominal surgery.

PN is a crucial replacement therapy of enteral nutrition in patients diagnosed with NEC. Literature has shown that patients usually needed PN for 2 weeks or even longer to support the life when EN was not available [41]. Our study also found that patients with CHD need more days of PN support and EN or oral feeding were started later, which may be due to the low perfusion of abdominal organs in the early postoperative stage of CHD [42]. To our limited knowledge, there are few studies focusing on the comparison of PN duration between CHD related NEC or preterm related NEC, thus it will be a new direction in our further study with a larger sample size.

This study has some limitations. Firstly, this study has a retrospective design and lacks data on maternal comorbidities during pregnancy, monitoring indicators, and certain interventions and treatments such as prostaglandin and probiotic treatments. While both hospitals are allied units and the treatment concepts are generally consistent, some monitoring indicators were absent due to differences in hardware equipment, such as near-infrared spectroscopy monitoring, CO monitoring, and bedside ultrasound. Secondly, the study did not provide a long-term follow-up of the children, which include data on growth and neurological development, the survival and quality of life of the children after discharge from the hospital.

## Conclusion

Compared with the nCHD-NEC group, the CHD-NEC group exhibited an older gestational age; higher birth weight; higher proportions of patients with PDA, shock, and the need for mechanical ventilation; and a higher VIS during onset but lower proportion of SGA; lower incidence of premature rupture of membranes, breastfeeding rate, frequency of RBC transfusions, and incidence of early- and late-onset sepsis. The main pathophysiological mechanism of CHD-NEC in children may be mesenteric ischemia or hypoperfusion. Therefore, accurate cardiac surgery timing, comprehensive circulatory management during the perioperative period, shock and anemia correction, and systemic circulatory perfusion increase may have a protective effect on the intestinal tract. Given that nCHD-NEC is mainly related to immature intestinal development and infection in preterm infants, breastfeeding should be encouraged during hospitalization, and the amount of feeding should be increased gradually. Effective anti-infection treatment and nosocomial infection prevention during NICU stay are crucial. Among children requiring abdominal surgery, those in the CHD-NEC group were more likely to have colon involvement, and there was no significant difference in mortality between the two groups.

## Data Availability

The datasets used and/or analyzed during the current study are available from the corresponding author on reasonable request.
